# Retraction Note: Restored microRNA-326-5p Inhibits Neuronal Apoptosis and Attenuates Mitochondrial Damage via Suppressing STAT3 in Cerebral Ischemia/Reperfusion Injury

**DOI:** 10.1186/s11671-022-03766-5

**Published:** 2022-12-09

**Authors:** Yumin Huang, Yingge Wang, Zuowei Duan, Jingyan Liang, Yijun Xu, Shuai Zhang, Tieyu Tang

**Affiliations:** 1 Department of Respiratory and Critical Medicine, Affiliated Hospital of Yangzhou University, Yangzhou University, Yangzhou, 225001 People’s Republic of China; 2 Department of Neurology, Affiliated Hospital of Yangzhou University, Yangzhou University, 45 Taizhou Road, Yangzhou, 225001 Jiangsu People’s Republic of China; 3 Institute of Translational Medicine, Medical College, Yangzhou University, Yangzhou, 225001 People’s Republic of China; 4 Jiangsu Key Laboratory of Integrated Traditional Chinese and Western Medicine for Prevention and Treatment of Senile Diseases, Yangzhou University, Yangzhou, 225001 People’s Republic of China; 5 Department of Jiangsu Key Laboratory of Experimental, Translational Non‑coding RNA Research, Yangzhou, 225001 Jiangsu People’s Republic of China; 6 Medical College, Yangzhou University, Yangzhou, 225001 People’s Republic of China

**Retraction Note to: Nanoscale Res Lett (2021) 16:63** 10.1186/s11671-021-03520-3

The Editors in Chief have retracted this article. After publication, concerns were raised concerning the appearance of Western blots and a possible similarity between the STAT3 bands in Fig. 5 b and e. The authors did not respond to requests for further clarification and did not supply raw images or the ethics permit. The editors, therefore, have lost confidence in the integrity of the article's findings. The editor was not able to obtain a current email address for Authors Shuai Zhang and Jingyan Liang. Authors have not responded to any correspondence from the editor or publisher about this retraction.

